# Folate and Vitamin B‐12 Status Is Associated With Bone Mineral Density and Hip Strength of Postmenopausal Chinese‐Singaporean Women

**DOI:** 10.1002/jbm4.10399

**Published:** 2020-09-02

**Authors:** Maria Kalimeri, Francesca Leek, Nan Xin Wang, Huann Rong Koh, Nicole C Roy, David Cameron‐Smith, Marlena C Kruger, Christiani Jeyakumar Henry, John J Totman

**Affiliations:** ^1^ Clinical Imaging Research Centre, Yong Loo Lin School of Medicine National University of Singapore Republic of Singapore; ^2^ Clinical Nutrition Research Centre, Singapore Institute for Food and Biotechnology Innovation Agency for Science, Technology and Research (A*STAR) Republic of Singapore; ^3^ Food Nutrition & Health Team AgResearch Grasslands Palmerston North New Zealand; ^4^ High‐Value Nutrition, National Science Challenge, Liggins Institute The University of Auckland Auckland New Zealand; ^5^ Riddet Institute Massey University Palmerston North New Zealand; ^6^ Department of Human Nutrition University of Otago Dunedin New Zealand; ^7^ Liggins Institute The University of Auckland Auckland New Zealand; ^8^ Singapore Institute for Clinical Sciences, Agency for Science Technology and Research (A*STAR) Republic of Singapore; ^9^ School of Health Sciences Massey University Palmerston North New Zealand

**Keywords:** BONE MINERAL DENSITY, FOLATE, HIP STRENGTH, POSTMENOPAUSE, VITAMIN B‐12

## Abstract

The role of micronutrients such as folate and vitamin B‐12 in bone quality has been widely studied with conflicting results. Ethnicity seems to play a large role on nutrient intake, as diet varies across cultures. In this study, we examined the relationships of BMD, proximal femur strength, and bone resorption with plasma folate and vitamin B‐12 in a cohort of 93 healthy postmenopausal women of Chinese‐Singaporean descent. The parameters examined were areal (aBMD) and volumetric BMD (vBMD) of the proximal femur and the third lumbar vertebra (L3), total body aBMD, proximal femur bending, compressive and impact strength indices (composite strength indices) and circulating levels of C‐telopeptide of type I collagen. Eighteen participants (19.4%) had aBMD in the osteoporotic range (osteoporosis group), 59 (63.4%) in the osteopenic range (osteopenia group), and the remaining 16 (17.2%) in the normal range (normal BMD group). Circulating folate levels were significantly higher in the normal BMD group compared with the osteoporosis group. Using linear regression analysis, we found that overall, aBMD and vBMD are positively associated with folate concentrations, whereas composite strength indices were positively associated with vitamin B‐12 concentrations. These findings support the existing literature and suggest a link between levels of circulating folate/vitamin B‐12 and BMD/bone strength in the cohort examined. Further investigation is needed to examine if individuals with inadequate circulating levels of these nutrients could decrease their risk for fragility fractures through better nutrition or vitamin supplementation. © 2020 The Authors. *JBMR Plus* published by Wiley Periodicals, Inc. on behalf of American Society for Bone and Mineral Research © 2020 The Authors. *JBMR Plus* published by Wiley Periodicals LLC on behalf of American Society for Bone and Mineral Research.

## Introduction

Osteoporosis is a bone disease that affects primarily postmenopausal women. It is caused by an imbalance between the formation and resorption of bone and is characterized by low BMD, deterioration of bone microstructure, and increased risk of fragility fractures.

Folate (vitamin B‐9) and cobalamin (vitamin B‐12) are two vitamins of the B‐complex that affect bone health both directly and indirectly, as deficiencies in these micronutrients have been linked to elevated homocysteine levels.^(^ ^1^ ^)^ High levels of circulating homocysteine have been shown in in vitro studies to cause collagen cross‐linking impairment,^(^ ^2^, ^3^ ^)^ as well as to impact osteoclast^(^ ^4^ ^)^ and osteoblast^(^ ^5^ ^)^ activity, leading to poor bone health and fragility fractures.^(^ ^6^, ^7^ ^)^ Supplementation with B‐complex vitamins, including folate and vitamin B‐12, lowered homocysteine levels in a group of older people, but interestingly did not improve bone turnover, as seen from levels of serum bone biomarkers of bone resorption (degradation products of type I collagen) and formation (bone‐specific alkaline phosphatase).^(^ ^8^ ^)^ Moreover, there is no clear consensus on the relationships of circulating folate and vitamin B‐12 levels with bone health. Studies have shown that folate is positively associated with BMD^(^ ^9^ ^)^ and bone strength,^(^ ^10^ ^)^ whereas others have shown that these relationships are nonexistent.^(^ ^11^, ^12^ ^)^ Similar results have been presented for vitamin B‐12, circulating levels of which have been shown to have a positive relationship with BMD^(^ ^13^ ^)^ and to be inversely related to fracture risk,^(^ ^14^ ^)^ whereas other studies did not conclude that these relationships are statistically significant.^(^ ^9^, ^11^, ^12^, ^15^ ^)^ These inconsistencies in results are attributed to differences in the design of individual studies, relative to the age, gender, and ethnicity of participants. Most research has been conducted in white cohorts or cohorts of mixed ethnicities, whereas such studies in Asian populations are sparse.^(^ ^16^ ^)^ Because Asians have different dietary and lifestyle habits than white populations and the proportion of fractures in Asia is expected to rise in the coming decades,^(^ ^17^, ^18^ ^)^ studies focusing on Asian populations are necessary to improve osteoporosis prevention and management.

BMD is routinely measured using DXA. DXA measures the combined areal BMD (aBMD) of trabecular and cortical bone and is the gold standard for assessing osteoporosis status.^(^ ^19^ ^)^ QCT is used to perform volumetric BMD (vBMD) measurements, but it is not standard practice for osteoporosis assessment. As a result, there is limited knowledge of any relationships between these two micronutrients and bone volume, microarchitecture, or BMD of the individual bone compartments (cortical, trabecular).

Therefore, the aim of this study was to examine the relationship of plasma folate and vitamin B‐12 levels with BMD, proximal femur strength, and bone resorption in a group of healthy, postmenopausal Chinese‐Singaporean women. BMD was examined at the third lumbar vertebra (L3) and the proximal femur, using DXA (aBMD) and QCT (aBMD, vBMD). Bone resorption was assessed by the levels of C‐telopeptide of type I collagen (CTx‐1). Based on previous studies, our hypothesis was that folate and B‐12 levels are positively associated with BMD, but not with bone resorption.

## Materials and Methods

### Cohort

Ninety‐seven healthy, postmenopausal Chinese‐Singaporean women were recruited from the general public at the Clinical Nutrition Research Centre, Singapore Institute for Food and Biotechnology Innovation (CNRC), through announcements on notice boards on the National University of Singapore campus and word of mouth. Inclusion criteria were BMI ranging between 18 and 30 kg/m^2^, age ranging between 55 and 70 years old, at least 5 years postmenopausal, in good general health with no diagnosis of conditions that affect bone metabolism (eg, osteoporosis, diabetes, arthritis, or cancer), and no recent fractures and renal or liver disease. Also, the participants should not smoke, consume less than two drinks containing 10ml or 8g of pure alcohol each, and in the 6 months prior to examination, they should not have been on medication that could affect bone health, such as glucocorticoids, hormone replacement therapy, and vitamin D or calcitonin. The study was given ethical approval by the National Healthcare Group; all the participants gave informed written consent prior to the examination. The study has been indexed in clinicaltrials.gov (NCT 03309254; Role of Glycaemic Index and High Protein Meal in Response of Blood Biomarkers for Pre‐diabetes).

### Anthropometric measurements

Anthropometric measurements were performed at CNRC. The participants provided information about their age and years since the onset of postmenopause. Height and weight were measured twice for each participant, and the average values were used to calculate BMI as the ratio of weight and squared height (kg/m^2^).

### Biochemical measurements

Fasting (12 hours overnight) and postprandial blood samples were collected and centrifuged at 1500*g* for 10 min at CNRC, and plasma aliquots were sent to the National University Hospital Singapore‐Referral Laboratory for analysis. The measurements we used for this analysis were fasting insulin, glucose, folate, and vitamin B‐12. Insulin was measured using the Cobas e411 (Roche Diagnostics, Mannheim, Germany). Glucose was measured using a photometric assay following the hexokinase method. Folate and vitamin B‐12 concentrations were measured using the competitive electrochemiluminescence immunoassay on the ADVIA Centaur Immunoassay system (Siemens Healthineers AG, Erlangen, Germany). Fasting plasma samples were also frozen, transferred to Massey University, New Zealand, and forwarded to Canterbury Health Laboratories, New Zealand, for analysis. There, PTH and CTx‐1 were measured by electrochemiluminescence immunoassay using the Roche COBAS e411 system (Roche Diagnostics). Then 25 (OH) vitamin D3 (vitamin D) was measured using isotope‐dilution liquid chromatography–tandem mass spectrometry. Insulin resistance status was assessed from the fasting insulin and fasting glucose levels, using the homeostasis model assessment for insulin resistance (HOMA‐IR): HOMA‐IR = (fasting insulin [mIU/L] · fasting glucose [mmol/L])/22.5, where higher values of HOMA‐IR indicate higher levels of insulin resistance.^(^ ^20^, ^21^, ^22^ ^)^

### Body composition and areal bone mineral density with DXA

Body composition measurements were performed at CNRC. Whole‐body fat mass (FM) and lean mass (LM), as well as aBMD of the whole body, the L1 to L4 vertebrae (each vertebra separately and of the whole lumbar spine), the femoral neck and total hip in both legs were measured by DXA (Hologic Discovery QDR 4500A densitometer; Hologic, Inc, Bedford, MA, USA). *T*‐scores were calculated for each site, and the participants were classified as in the normal, osteopenic, or osteoporotic range, according to the lowest *T*‐score value of the lumbar spine, femoral neck, and total hip of both legs.^(^ ^23^ ^)^

### Bone mineral density and femur‐size measurements with QCT

vBMD measurements were performed at the Clinical Imaging Research Centre, Yong Loo Lin School of Medicine, National University of Singapore. Volumetric QCT of both proximal femurs and the L3 was performed in a clinical CT scanner (Siemens mCT, Erlangen, Germany), using a commercial phantom (Mindways Software, Inc, Austin, TX, USA). Peak kilovoltage was 120 kVp and tube current was 105 mAs for the femurs and 53 mAs for the L3. The scans were analyzed using the phantom's standard software (QCT Pro, version 5.0). vBMD measurements of L3 were performed, as well as of the whole bone and the trabecular and cortical compartments of the femoral neck and the total hip of the nondominant leg. Additionally, femoral neck and total hip aBMD of the whole bone and the separate bone compartments, femoral neck width (FNW), and hip axis length (HAL) were calculated from projections of the proximal femur.

### Femoral neck strength

Proximal femur strength was assessed using composite strength indices.^(^ ^24^, ^25^ ^)^ These indices use aBMD of the femoral neck, geometric characteristics of the proximal femur (FNW, HAL), and the weight and height of the participant to estimate femoral strength under compressive, bending, and impact forces as follows.

$$\text{Compression strength index}\;\left(\mathrm{CSI}\right)=\frac{\text{aBMD}∙\mathrm{FNW}}{\text{Weight}}$$

$$\text{Bending strength index}\;\left(\mathrm{BSI}\right)=\frac{\text{aBMD}∙{\mathrm{FNW}}^{2}}{\mathrm{HAL}∙\text{Weight}}$$

$$\text{Impact strength index}\;\left(\mathrm{ISI}\right)=\frac{\text{aBMD}∙\mathrm{FNW}∙\mathrm{HAL}}{\text{Height}∙\text{Weight}}$$

### Statistical analysis

Statistical analysis was performed using SPSS for Windows, version 25 (SPSS, Inc, Chicago, IL, USA). Levene's test was used to assess homogeneity of variance in groups. Differences between the three groups were examined by means of one‐way ANOVA. Post hoc tests performed for the parameters with statistically significant ANOVA results; the Hochberg and Games‐Howell post hoc tests were used in the homogenous and the nonhomogenous groups, respectively. Multivariate regression analysis was performed to assess the linear relationships among the BMD measurements, composite strength indices, and CTx‐1 with plasma folate and vitamin B‐12. Age, BMI, FM, LM, PTH, vitamin D, and HOMA‐IR were also included as independent variables. Because QCT vBMD of the lumbar spine was only measured at the L3, DXA aBMD of the L3, instead of the L1 to L4, was also used as a dependent variable to compare the measurements of the same location made using different modalities. The natural log (ln) transformation was applied to folate, vitamin B‐12, and HOMA‐IR values because they were not normally distributed parameters. As the predictors included in the regression model have been shown to affect bone quality, we assumed that the *R* ^2^ of the model will vary between 0.15 and 0.3 for different dependent parameters, which means that the effect size of the model would range from *f* ^2^ = 0.13 (medium effect) to *f* ^2^ = 0.4 (large effect), respectively. Using the software package G*Power^(^ ^26^ ^)^ with α = 0.05, this model with 9 predictors and sample size of between 91 and 93 is expected to have a level of power between 68% and 99%. Between‐group differences and parameter contribution to the linear regression models were considered statistically significant when *p* < 0.05.

## Results

### Cohort characteristics

The average age of the participants was 60.8 (±4.2) years old, and average BMI was 22.9 (±2.6) kg/m^2^. Fasting glucose levels for one of the participants were above the normal level (>7 mmol/L); this participant was excluded from further analysis. Participants with values that are extreme outliers (>3 SDs from the mean) were also excluded. One participant had an extreme PTH measurement; one participant had an extreme vitamin B‐12 measurement; and one participant had extreme CTX‐1 and PTH measurements. These extreme values could not be explained; we are not aware if they were caused by measurement error, wrong user input, or an undetected underlying health condition,^(^ ^27^ ^)^ thus they were considered nonrepresentative of the population. Of the 93 remaining participants, 18 (19.4%) had aBMD in the osteoporotic range (osteoporosis group), 59 (63.4%) in the osteopenic range (osteopenia group), and the remaining 16 participants (17.2%) were in the normal range (normal BMD group). The average values of BMD for the cohort, as well as of each group separately, are shown in Table 1. vBMD measurements of the proximal femur were invalid for one of the participants because of the incorrect automatic placement of the regions of interest by the phantom's standard software, and FNW and HAL measurements were not available for an additional participant. The average values of folate and vitamin B‐12, in the whole cohort and in each separate group, indicated sufficient circulating levels of both micronutrients.^(^ ^28^ ^)^ Participants with missing values were excluded from the linear regression analysis. As a result, composite strength indices were examined for 91 participants, volumetric measurements of the proximal femur (QCT) for 92 participants, and all DXA measurements, volumetric measurements of the L3 (QCT), and CTx‐1 for 93 participants. Because FNAL and HAL were measured by means of QCT, QCT aBMD measurements of the whole bone, which consists of both the cortical and trabecular compartments, were used for the calculation of the composite strength indices.

**Table 1:** aBMD and vBMD Measurements of the Whole Cohort and Each Group Separately

	All subjects (*n* = 93)	Osteoporosis (*n*_1_ = 18)	Osteopenia (*n*_2_ = 59)	Normal (*n*_3_ = 16)
DXA aBMD (g/cm^2^)				
Femoral neck	0.64 ± 0.09	0.53 ± 0.04	0.64 ± 0.05	0.77 ± 0.05
Total hip	0.79 ± 0.10	0.66 ± 0.05	0.79 ± 0.07	0.92 ± 0.07
L3 vertebra	0.88 ± 0.15	0.75 ± 0.11	0.88 ± 0.13	1.03 ± 0.11
Total body	0.96 ± 0.08	0.88 ± 0.04	0.97 ± 0.07	1.05 ± 0.07
QCT aBMD (g/cm^2^)				
Femoral neck^a^				
Whole	0.68 ± 0.11	0.57 ± 0.08	0.68 ± 0.07	0.80 ± 0.11
Cortical	0.44 ± 0.10	0.35 ± 0.08	0.43 ± 0.06	0.54 ± 0.13
Trabecular	0.24 ± 0.04	0.22 ± 0.04	0.24 ± 0.03	0.25 ± 0.04
Total hip^a^				
Whole	0.74 ± 0.11	0.61 ± 0.06	0.74 ± 0.08	0.87 ± 0.08
Cortical	0.45 ± 0.10	0.35 ± 0.05	0.45 ± 0.08	0.56 ± 0.10
Trabecular	0.28 ± 0.03	0.26 ± 0.03	0.29 ± 0.03	0.31 ± 0.03
QCT vBMD (g/cm^3^)				
Femoral neck^a^				
Whole	309.06 ± 50.27	256.96 ± 38.81	309.07 ± 30.65	364.38 ± 61.27
Cortical	986.15 ± 110.29	1090.31 ± 128.97	976.12 ± 94.50	912.45 ± 55.10
Trabecular	137.79 ± 19.58	116.81 ± 14.80	138.62 ± 15.24	157.01 ± 17.28
Cortical to total volume ratio	0.21 ± 0.06	0.15 ± 0.05	0.21 ± 0.04	0.28 ± 0.08
Total hip^a^				
Whole	275.36 ± 52.01	232.67 ± 29.77	274.49 ± 47.90	323.94 ± 44.81
Cortical	923.75 ± 78.03	991.04 ± 79.52	915.84 ± 74.99	881.43 ± 34.09
Trabecular	132.42 ± 17.51	113.97 ± 11.91	132.88 ± 14.09	150.31 ± 14.61
Cortical to total volume ratio	0.19 ± 0.05	0.14 ± 0.04	0.19 ± 0.04	0.24 ± 0.06
L3 vertebra^b^				
Trabecular	105.95 ± 25.38	85.89 ± 18.62	105.68 ± 23.84	129.54 ± 16.82

aBMD = Areal bone mineral density; vBMD = volumetric bone mineral density.

^a^ *n* = 93.

^b^ *n* = 92.

### Statistical analysis

The independent variables used in linear regression analysis, their average values, and SDs, as well as *p* values resulting from one‐way ANOVA are presented in Table 2. Post hoc tests of the data with *p* < 0.05 in ANOVA showed the following; BMI was significantly lower in the osteoporosis group than the normal BMD group (*p* < 0.01) and LM was significantly lower in the osteoporosis group than the normal BMD group (*p* = 0.03). Finally, folate levels were higher in the normal BMD group than in the osteoporosis group (*p* = 0.04).

**Table 2:** Average Values and Standard Deviations of the Anthropometric and Laboratory Findings Examined for the Whole Group and the Three Subgroups (Normal, Osteopenic, Osteoporotic Range) and Significance Levels (*p* Values) of One‐Way ANOVA

	Cohort (*n* = 93)	Osteoporosis (*n*_1_ = 18)	Osteopenia (*n*_2_ = 59)	Normal (*n*_3_ = 16)
Age (years)	60.8 ± 4.2	62.3 ± 4.0	60.8 ± 4.2	59.1 ± 4.0
BMI (kg/m^2^)	22.9 ± 2.6	^a^21.6 ± 2.0	23.0 ± 2.7	23.9 ± 2.8
Fat mass (kg)	21.8 ± 4.9	20.0 ± 3.7	22.0 ± 5.0	22.8 ± 5.3
Lean body mass (kg)	31.6 ± 3.1	^a^30.1 ± 2.7	31.7 ± 3.2	32.8 ± 2.8
25(OH) D (nmol/L)	59.9 ± 14.5	58.8 ± 14.4	60.0 ± 14.2	61.3 ± 16.3
PTH (pmol/L)	4.6 ± 1.3	4.5 ± 1.4	4.6 ± 1.3	4.8 ± 1.2
Folate (nmol/L)	35.6 ± 17.2	^a^28.8 ± 14.9	35.6 ± 15.8	43.2 ± 21.7
Vitamin B‐12 (ng/mL)	313.6 ± 139.4	277.1 ± 121.5	313.5 ± 120.3	343.9 ± 186.8
HOMA‐IR	1.34 ± 0.71	1.32 ± 0.77	1.33 ± 0.57	1.41 ± 0.70

HOMA‐IR = Homeostasis model assessment for insulin resistance.

^a^ Significantly lower than the normal BMD group.

Table 3 presents the results of linear regression analysis with bone‐related measurements as dependent parameters and the parameters listed in Table 2 as independent parameters. The results are presented as standardized β coefficients and *p* values of ln(folate) and ln(vitamin B‐12). It should be noted that because folate and vitamin B‐12 values were log‐transformed, the standardized β coefficients do not show a direct biological effect, but are an indication of the direction and magnitude of the relationship between the dependent and independent parameters. The results of regression analysis for age, BMI, FM, LM, vitamin D, PTH levels, and ln(HOMA‐IR) as independent parameters are not shown in Table 3, but can be found in Supplementary Table S1. It should be noted that for two dependent parameters (trabecular QCT aBMD of the femoral neck and the total hip), *R* ^2^ was smaller than our assumed 0.15, and in those cases the statistical power of the model is lower than 68%.

**Table 3:** Results of Multivariate Linear Regression Analysis: Standardized β Coefficients and *p* Values for Independent Parameters ln(Folate) and ln(Vitamin B‐12)

	*R* ^2^	Ln(folate)		Ln(B‐12)	
		β	*p* Value	β	*p* Value
DXA aBMD^a^					
Femoral neck	0.286	0.243	0.02^*^	0.143	0.16
Total hip	0.259	0.336	0.00^**^	0.075	0.47
L3 vertebra	0.208	0.149	0.16	0.011	0.92
Total body	0.296	0.237	0.02^*^	0.055	0.59
QCT aBMD					
Femoral neck^b^					
Whole	0.280	0.199	0.05	0.184	0.08
Cortical	0.221	0.180	0.09	0.142	0.19
Trabecular	0.106	0.060	0.60	0.185	0.11
Total hip^b^					
Whole	0.249	0.317	0.00^**^	0.085	0.42
Cortical	0.238	0.302	0.00^**^	0.042	0.69
Trabecular	0.137	0.140	0.21	0.160	0.16
QCT vBMD					
Femoral neck^b^					
Whole	0.209	0.265	0.02^*^	0.092	0.40
Cortical	0.193	−0.161	0.14	−0.147	0.18
Trabecular	0.172	0.259	0.02^*^	0.140	0.21
Cortical to total volume ratio	0.227	0.213	0.05	0.092	0.39
Total hip^b^					
Whole	0.149	0.247	0.03^*^	−0.017	0.88
Cortical	0.208	−0.259	0.02^*^	0.109	0.32
Trabecular	0.166	0.317	0.00^**^	0.108	0.33
Cortical to total volume ratio	0.225	0.299	0.01^*^	0.020	0.85
L3 vertebra^a^					
Trabecular	0.195	0.220	0.04^*^	0.081	0.45
Composite strength indices^c^					
CSI	0.349	0.028	0.77	0.226	0.03^*^
BSI	0.251	−0.092	0.38	0.272	0.01^*^
ISI	0.352	0.033	0.74	0.176	0.08
Bone resorption marker^a^					
CTx‐1	0.152	−0.202	0.07	0.014	0.90

aBMD, vBMD, cortical to total‐bone ratio, composite strength indices, and CTX‐1 were the dependent values in the model. The results for the other independent parameters used in the model listed in Table 2 are shown in Supplementary Material Table S1. aBMD = areal bone mineral density; BSI = bending strength index; CSI = compression strength index; CTx‐1 = C‐telopeptide of type I collagen; ISI = impact strength index; vBMD = volumetric Bone mineral density; β = standardized coefficient β.

^a^ *n* = 93.

^b^ *n* = 92.

^c^ *n* = 91.

^*^ *p* < 0.05.

^**^ *p* < 0.01.

### Areal bone mineral density with DXA

Analysis of aBMD results measured by means of DXA shows that total body, femoral neck, and total hip aBMD are positively associated with folate concentrations. The association of folate with aBMD at the total hip was stronger than at the femoral neck. The relationship between L3 aBMD and folate was not significant at the *p* = 0.05 level.

### Bone mineral density measurements with QCT

QCT measurements of aBMD (referred to as QCT aBMD) were only performed at the proximal femur. Analysis showed that QCT aBMD of the whole bone and the cortical compartment are positively associated with folate concentrations. At the femoral neck, these associations approximated significance (*p* = 0.05 and *p* = 0.09, for the whole and cortical compartment, respectively), whereas they reached statistical significance at the total hip (*p* < 0.05). Trabecular QCT aBMD was not associated with folate at a statistically significant level.

QCT vBMD at the proximal femur showed statistically significant associations between folate and vBMD at the femoral neck and the total hip, but the relationships were not consistent with those between QCT aBMD and folate. At the femoral neck, the whole bone and the trabecular compartment showed a strong positive association with folate (*p* < 0.05), and all vBMD measurements of the total hip, including the trabecular compartment, were also positively associated with folate (*p* < 0.01). Interestingly, the relationship between folate and cortical vBMD at the total hip was inverse (β < 0). Finally, the ratio of cortical to whole bone in both the femoral neck and the total hip was also positively associated with folate. QCT volumetric measurements of the trabecular compartment of L3 had a positive and statistically significant association with folate (*p* < 0.05).

### Composite strength indices

Composite strength indices of the proximal femur were not associated with folate at a statistically significant level, but had a positive relationship with vitamin B‐12. The relationship of vitamin B‐12 with CSI and BSI was statistically significant (*p* < 0.05), whereas with ISI, it approximated significance (*p* = 0.08).

### Bone resorption

Bone resorption marker CTx‐1 was shown to be inversely (β < 0) and marginally (*p* = 0.07) associated with folate, but not associated with vitamin B‐12 in this regression model.

## Discussion

To the best of our knowledge, this is the first study to investigate the relationship of plasma folate and vitamin B‐12 concentrations with aBMD and vBMD, proximal femur strength, and bone resorption in healthy postmenopausal Chinese‐Singaporean women. Additionally, we examined these relationships for whole‐bone measurements, as well as the different compartments individually. We concluded that circulating folate is positively associated with proximal femur aBMD and vBMD, total‐body aBMD, and L3 vBMD; circulating vitamin B‐12 levels correlated positively with bending and compression strength indices of the proximal femur.

The roles of folate and vitamin B‐12 deficiencies in osteoporosis have been investigated for their connection to increased homocysteine levels, as well as for their possible direct effect on BMD and fracture risk. High levels of plasma homocysteine have been shown to affect the cross‐linking of collagen and thus to compromise bone strength.^(^ ^29^ ^)^ The direct effect of folate and vitamin B‐12 deficiencies on the equilibrium of bone turnover has also been investigated; vitamin B‐12 deficiency has been shown to suppress osteoblast activity,^(^ ^30^ ^)^ whereas an experimental study has shown increased resorption activity in folate‐deficient osteoclasts compared with folate‐treated cells.^(^ ^31^ ^)^ Observational studies have not been consistent in finding the effects of elevated homocysteine, folate, or vitamin B‐12 deficiency directly on BMD and fracture risk.^(^ ^32^ ^)^ Such inconsistencies could be attributed to several factors, such as different cohorts and differences in study design and analysis. Interestingly though, unrelated studies have suggested that in the presence of elevated homocysteine levels, low folate concentrations are associated with low BMD and increased fracture risk. First, in a cohort with elevated homocysteine levels caused by a homozygous mutation of the gene methylenetetrahydrofolate reductase, proximal femur BMD was associated with serum folate concentration.^(^ ^33^ ^)^ Similarly, in a population study among elderly Italians, high homocysteine levels and low serum folate are a significant risk factor for osteoporotic fractures.^(^ ^10^ ^)^ Meanwhile, in a cohort of elderly Dutch men and women, it was found that women, but not men, with osteoporosis had lower plasma vitamin B‐12 than their peers with normal BMD or osteopenia, although plasma homocysteine levels were not significantly different between the three groups.^(^ ^34^ ^)^ Finally, in a cohort of British postmenopausal women,^(^ ^35^ ^)^ it was found that participants with osteoporosis had significantly lower serum folate than participants with osteopenia or normal aBMD at the calcaneus, whereas serum vitamin B‐12 did not differ significantly among the three groups. In the same study, serum folate and homocysteine were found to be associated with aBMD, but this relationship was not significant for homocysteine after taking vitamin B‐6, folate, and vitamin B‐12 into account. These findings suggest a direct role for folate and vitamin B‐12 in the protection of bone health.

DXA is the most common method used in studies for measuring aBMD because of its ease of use, low cost, and low radiation exposure. QCT is not widely used for measuring BMD, but it has been shown to produce more accurate results in osteoporosis classification^(^ ^36^ ^)^ because the 3D nature of QCT allows the calculation of BMD, ignoring other overlaying tissues unrelated to bone (eg, aortic calcifications), as well as that of separate compartments. The latter can offer insight into whether changes occur in only one compartment, which is not possible with DXA. Here, we will discuss the relationship of folate with all whole‐bone measurements (DXA and QCT aBMD, QCT vBMD) in the proximal femur and the L3. Our hypothesis was that both folate and vitamin B‐12 are positively associated with aBMD and vBMD, but our analysis showed that, in our cohort, this association was statistically significant only for folate. As already mentioned, there is no clear consensus on the relationship of folate and vitamin B‐12 to BMD, but our observations are in agreement with the results of a study on postmenopausal Italian women,^(^ ^9^ ^)^ where folate, but not vitamin B‐12 or homocysteine, was also found to be associated with BMD and standardized 12‐month changes in lumbar spine BMD (L2 to L4). A difference between the results of the two studies is that we found aBMD of the proximal femur and the total body to be significantly associated with folate, but not aBMD of the lumbar spine (L3). This could be attributed to the cohort composition of each study (Asian and white), which comes with different dietary and lifestyle habits. Instead, this association was statistically significant for L3 vBMD in our cohort, although it should be noted that L3 vBMD was measured for the trabecular compartment only, which could explain this discrepancy in findings within our cohort. In another study, conducted on a cohort of older (>50 years old) white, black, and Mexican‐American women in the United States found a positive linear association of both folate and vitamin B‐12 concentrations with aBMD of the L3 and the total body.^(^ ^37^ ^)^ Food fortification with folate in the United States led to similar folate levels across BMD groups in the US study, whereas there were differences between groups in our study (Table 2). These differences in folate concentrations, together with the younger age of participants in the US study, could be reasons behind the differences in the results between this study and ours. Another study of elderly men and women, also conducted in the United States, concluded that BMD was associated with serum vitamin B‐12 only at lower concentrations (100 to 200 pmol/L),^(^ ^38^ ^)^ an effect also observed in a categorical analysis of data from the Framingham Osteoporosis Study.^(^ ^13^ ^)^ The US study only examined the effect of vitamin B‐12 on BMD independently of homocysteine, and also showed that men with adequate levels of vitamin B‐12 (>350 pg/mL) had higher proximal femur BMD than men with inadequate levels of vitamin B‐12 (<200 pg/mL), whereas the differences between these two categories in women were significant at the lumbar spine and all sites of the proximal femur, but not the femoral neck. The participants in our study had adequate concentrations of vitamin B‐12, and this, possibly together with the differences in the cohort characteristics, could explain why in our study the association between BMD and vitamin B‐12 concentrations was not statistically significant. Finally, a study of younger postmenopausal Turkish women^(^ ^39^ ^)^ found that lumbar BMD was not associated with folate or vitamin B‐12, and that there is no difference in plasma folate concentrations between the normal BMD group and the osteoporosis and osteopenia groups. Overall, our results suggest that in our cohort and using our regression model, there are positive associations between BMD measurements and folate in a manner similar to existing literature, and for adequate levels of circulating vitamin B‐12, there is no association between vitamin B‐12 and BMD.

The separate bone compartments play complementary roles in bone strength and osteoporosis affects each compartment differently.^(^ ^40^ ^)^ Thus, we used QCT to measure the aBMD and vBMD of separate compartments of bone in the proximal femur and investigated possible associations of each with folate and vitamin B‐12. We found that folate was significantly associated with cortical, but not trabecular QCT aBMD of the total hip. Neither cortical nor trabecular QCT aBMD at the femoral neck was associated with folate. In an in vitro study of femoral heads extracted from men and women undergoing hip arthroplasty,^(^ ^41^ ^)^ histological parameters of trabecular bone showed superior trabecular microarchitecture in individuals with higher folate concentrations, although there were no differences in aBMD between people with low and high folate or vitamin B‐12. Thus, in our study, lower folate concentration could also be associated with the structural properties rather than in the actual mineralization of bone, and as a result, cannot be detected as a statistically significant association with aBMD levels. This is a very interesting hypothesis, as the structural properties of trabecular bone contribute largely to bone strength, but this is not necessarily reflected on BMD measurements. On the contrary though, vBMD measurements of the separate compartments of the proximal femur showed that trabecular vBMD of the femoral neck and the total hip is significantly and positively associated with folate concentrations. Studies on the relationships between vBMD and vitamins of the B‐complex are limited, and we are not aware of any studies examining the proximal femur or the lumbar spine. One study on healthy postmenopausal women in the United States though,^(^ ^42^ ^)^ reported no statistically significant associations between folate or vitamin B‐12 and trabecular vBMD or BMC in the distal tibia. Finally, we found that the association of circulating folate with cortical vBMD remained nonsignificant at the femoral neck, but it was statistically significant and negative at the total hip. This relationship is in contrast to the positive association between cortical QCT aBMD discussed above, and to our knowledge, has not been reported elsewhere; thus, further investigation is needed to examine its validity. Our results regarding possible associations between the two separate bone compartments and folate and vitamin B‐12 were inconclusive, as aBMD and vBMD measurements did not follow similar trends.

Composite strength indices of the proximal femur and cortical‐ to total‐bone‐volume ratios were used as proxies for bone strength and fracture risk in the present study. The ratio between cortical bone and whole bone in the total hip was positively associated with the concentration of folate, which suggests that the cortical shell in this anatomical site is thicker at higher concentrations of folate. This finding though, combined with the inconclusive result of the relationship between cortical BMD (aBMD and vBMD) discussed earlier, does not allow us to reach a plausible conclusion regarding the meaning of thicker cortical shell for higher folate concentrations. Composite strength indices of the proximal femur were not associated with folate concentration, but were instead positively associated with vitamin B‐12 concentrations. More specifically, CSI and BSI were positively associated with vitamin B‐12, whereas the same association for ISI followed the same trend, but was only marginally significant. This finding is in agreement with results of a meta‐analysis study,^(^ ^32^ ^)^ where a stepwise increase in vitamin B‐12 concentrations was associated with a decrease in fracture risk. On the other hand, contrary to our findings, in a population study among Chinese‐Singaporeans, no associations of dietary folate and vitamin B‐12 with fracture risk, assessed through fragility fractures suffered by participants within an average follow‐up of 13.8 years, were found.^(^ ^16^ ^)^ It should be pointed out that the present study and Dai et al.'s study^(16^ ^)^ cannot be directly compared because fragility fracture risk and vitamin B‐12 levels were estimated differently. The aim of osteoporosis screening is to reduce fragility fractures, so a better understanding of fragility fracture risk and ways to decrease it are of utmost importance, and composite strength indices of the proximal femur could be investigated as a method to assess this risk. Additionally, further research is required to conclude whether these indices can detect possible changes in bone strength based on interventions—nutritional or otherwise.

Bone turnover markers can indicate changes in bone metabolism in a shorter period than measurements of BMD. As a result, the efficacy of an intervention can be studied at its early stages by measuring bone turnover, instead of waiting to detect possible changes in BMD. Our analysis showed an inverse, albeit of marginal statistical significance, association between circulating levels of the bone resorption marker CTx‐1 and folate. Higher levels of bone resorption indicate faster bone loss, and thus decreased levels of BMD. Consequently, this association, although only marginally significant, is in agreement with the overall association of folate and BMD that we observed in our cohort. Contrary to our findings, it has been reported^(^ ^39^ ^)^ that in younger postmenopausal Turkish women (postmenopausal for over a year), there was no association between CTx‐1 and serum folate or vitamin B‐12. It has been shown that bone resorption reaches its peak in 5 to 10 years after the onset of postmenopause compared with the years before and after that timeframe.^(^ ^43^ ^)^ In the aforementioned study,^(^ ^39^ ^)^ the women were postmenopausal for at least a year, whereas in our study, for at least 5 years. Thus, we could argue that the observed relationship between folate and CTx‐1 in these two studies was affected by the years since the onset of postmenopause, as well as possible differences in diet, considering that the two cohorts resided in different geographic locations. This negative trend between CTx‐1 and circulating folate should be further investigated, likely in studies involving a larger population, to reach a more definitive conclusion.

This study examined the relationship of bone health with folate and vitamin B‐12 in a homogenous cohort of postmenopausal women with a wide range of BMDs. The use of QCT, in addition to DXA, allowed us to examine possible associations between these micronutrients and volumetric measurements, as well as the aBMD and vBMD of different compartments of the bone. Finally, the sites examined (proximal femur, L3), are of clinical importance, but are not commonly assessed with QCT. On the other hand, because folate and vitamin B‐12 are closely linked with homocysteine, it would have been interesting to account for its effect, also possibly making comparisons to other studies more straightforward. Moreover, data on bone formation markers, which were also not available, would have given an insight into whether bone formation is associated with plasma folate and vitamin B‐12. Finally, as folate and vitamin B‐12 supplementation were not among the exclusion criteria, it is not certain if any of the participants consumed supplements containing these vitamins. Such information would allow to differentiate the effect of supplementation, but we believe that it does not influence the conclusions of the current analysis.

To the best of our knowledge, this is the first study investigating associations between plasma folate and vitamin B‐12 levels and bone‐quality characteristics in postmenopausal Chinese‐Singaporean women. Both areal and volumetric imaging technologies were employed to measure BMD, whereas bone resorption was assessed from plasma CTx‐1 levels and composite strength indices were used to approximate bone strength. We found that, overall, BMD was associated with plasma folate levels, whereas proximal femur strength, under conditions of bending and compression, was positively related to plasma vitamin B‐12 concentrations. Considering that fragility fractures in Asian populations are expected to rise in the coming decades and that nutrition is an easily modifiable factor that affects bone health, the management and prevention of osteoporosis in the region would benefit from further studies.

## Disclosures

All authors state that they have no conflicts of interest.

## AUTHOR CONTRIBUTIONS

**Maria Kalimeri:** Formal analysis; methodology; writing‐original draft; writing‐review and editing. **Francesca Leek:** Data curation. **Nan Xin Wang:** Data curation. **Huann Rong Koh:** Data curation. **Nicole Roy:** Conceptualization. **David Cameron‐Smith:** Conceptualization. **Marlena Kruger:** Conceptualization; data curation. **Christiani Jeyakumar Henry:** Conceptualization; data curation. **John Totman:** Conceptualization; data curation.

## Supporting information


**Table S1.** Results of multivariate linear regression analysis: standardized β coefficients and *p* values for all independent parameters used in the study. aBMD, vBMD, cortical to total bone ratio, composite strength indices and CTX‐1 were the dependent values in the model.
